# Inhibition of HMGB1/RAGE axis suppressed the lipopolysaccharide (LPS)-induced vicious transformation of cervical epithelial cells

**DOI:** 10.1080/21655979.2021.1957750

**Published:** 2021-08-08

**Authors:** Lifang You, Hongyin Cui, Fen Zhao, Huier Sun, Huanxin Zhong, Guoli Zhou, Xuejun Chen

**Affiliations:** a Department of Gynecology, First People’s Hospital of Yuhang District, Hangzhou, China; b Laboratory Department, First People’s Hospital of Yuhang District, Hangzhou, China

**Keywords:** Inflammation, normal cervical epithelial cells, HMGB1/RAGE axis, malignant transformation

## Abstract

The chronic inflammation operates as one of the critical causes of cervical cancer. Activation of HMGB1/RAGE axis could induce the inflammation and lead to multiple types of cancer. However, whether the HMGB1/RAGE axis could affect the development of cervical cancer by regulating the inflammation is unclear. Here, we stimulated normal cervical epithelial cells with lipopolysaccharide (LPS). Next, the expression of RAGE in these cells was suppressed by the RAGE inhibitor. CCK-8 and wound healing assays were performed to detect the proliferation and invasion. To determine how inflammatory factors (IL-1β, IL-6 and TNF-α) expressed in supernatant of these cells, ELISA was conducted. Western blotting was used for the detection of the expression of pyroptosis-related proteins (NLRP3 and caspase4). It was found that stimulation of LPS enhanced the proliferation and invasion of normal cervical epithelial cells. The expression of inflammatory factors (IL-1β, IL-6 and TNF-α) in these cells was promoted as well. Application of RAGE inhibitor abolished the efficacy of LPS on these cells. Furthermore, LPS promoted the expression of NLRP3 and caspase4 in these cells while RAGE inhibitor exerted suppressive effects on the expression of these proteins. In summary, LPS-induced inflammation of normal cervical epithelial cells resulted in the malignant transformation of these cells by activating HMGB1/RAGE axis.

## Introduction

Cervical cancer is known as a malignant tumor that commonly occurs in gynecology. Increasing morbidity and mortality of cervical cancer have been witnessed in recent years, putting tremendous pressure on the public health affairs worldwide [1]. Moreover, the strong metastasis of cervical cancer cells increased the risk of recurrence. Simultaneously, patients with cervical cancer suffered many hardships during prognosis [2]. Therefore, more efforts should be put into exploring the molecular mechanism of the occurrence and development of cervical cancer, in order to discover more targeted therapies.

The occurrence of chronic inflammation has always been considered a crucial cause of tumor [3]. The progression of cancer is characterized by inflammation. The production of pro-inflammatory factors plays a crucial role in the development of advanced cancer [4]. In addition, the inflammatory response is associated with the pathology of the tumor. The increasing expression of hyperactivation of nuclear factor-κB (NF-κB) also enhanced the metastasis and proliferation of various tumor cells [5]. Moreover, the infection of human papilloma virus (HPV) is the significant cause of the occurrence and development of cervical cancer [6]. Most transient HPV infections could be cured by the human immune system, while continuous infections could end in cervical cancer [7]. In patients with persistent HPV infection, the expression of inflammatory factors was increasing significantly, and these inflammatory reactions ultimately led to cervical cancer [8]. Furthermore, the inflammatory diseases induced by trichomoniasis, bacteria and even fungi can also give rise to malignant proliferation and canceration of normal cells in the cervix [9]. Therefore, the detection of these causative agents, such as Nuoliqing commercial kits which is widely used for detecting trichomoniasis, bacteria and fungi, is critical for the prevention of infection-induced cervix uteri inflammation.

The HMGB1 (High mobility group box 1, HMGB1) is a DNA-bound nuclear protein which is actively released after cytokine stimulation and passively released after the death of these cells [10]. Advanced glycation end product receptor (RAGE) is a receptor with many ligands that could bind molecules of diverse structures [11]. Existing studies suggested that the expression of HMGB1 could promote the occurrence and development of inflammation by activating the expression of RAGE [12]. RAGE blockage has inhibitory effects on cellular inflammation and apoptosis so as to achieve the prevention against cellular injury in various diseases [13,14]. However, whether the HMGB1/RAGE axis regulated the inflammation and occurrence of cervical cancer remained unknown.

Therefore, we aim to investigate the role of HMGB1/RAGE axis in chronic inflammation of cervical epithelial cells and to clarify its potential mechanism. In this study, cervical epithelial cells were stimulated with lipopolysaccharide (LPS) to induce chronic inflammation. Next, we determined the occurrence of the inflammation and clarified the effect of HMGB1/RAGE axis in this process. The results of our study might provide a new therapy for the clinic treatment of cervical cancer.

## Material and methods

### Cell culture and treatment

The ATCC (Manassas, VA, USA) was the provider for HCE cell lines (End1/E6E7 cells). Next, these cells were cultured with the RPMI1640 medium containing 10% fetal bovine serum (Gibco, USA). Then, cells underwent growth in a humid atmosphere with 5% CO_2_, and the temperature was controlled at 37°C. After that, we applied LPS (Thermo Fisher Scientific, USA) to stimulate these cells. In this process, four levels of LPS concentrations (0.1, 0.5, 1 and 5 μg/ml) were selected. Also, RAGE inhibitor FPS-ZM1 (Thermo Fisher Scientific, USA) was utilized to treat these cells.

### CCK-8 assays

Cell proliferation ability was detected using Cell Counting Kit-8 (CCK-8; Biosharp, Hefei, China). Briefly, cells were placed into the 96-well plate. After the adhesion of these cells, CCK-8 solution was diluted with the culture medium and added into these wells. In the following procedure, the plate was kept in the incubator for 1 hour. Finally, the absorbance was detected at 450 nm with a microplate reader (Thermo Fisher Scientific, USA).

### Wound healing assays

Cells were cultured in the 6-well plate. After the adhesion of these cells, serum-free medium was used for the cell culture which lasted for 12 hours. Then, we created the scratch on the bottom of these cells. The scratch was photographed with the microscope (Olympus, Japan). The following photograph of the scratch was taken after 24 hours. Finally, the width of the scratch was measured with the Image J (National Institutes of Health, USA).

### Flow cytometry assays

Flow cytometry was performed to determine cell apoptosis and cell cycle. For cell apoptosis assay, an Annexin-V-FITC Apoptosis Detection Kit (Beyotime, China) was adopted according to the manufacturer’s instructions. Briefly, cells were collected by trypsinization and washed with PBS. Subsequently, cells were suspended with binding buffer and incubated with Annexin V and PI for 15 min in the dark. Cell apoptosis rate was analyzed by flow cytometry using a FACScan flow cytometer (BD Biosciences, San Jose, USA). For cell cycle assay, cells were collected by trypsinization, fixed with 70% ice-cold ethanol, and washed with PBS. Subsequently, cells were suspended with binding buffer and incubated with RNase and PI for 30 min. Finally, Cell apoptosis rate was analyzed by flow cytometry using a FACScan flow cytometer (BD Biosciences, San Jose, USA).

### ELISA assays

The supernatant of these cells was collected with the tube. Then Human Interleukin (IL)-1β ELISA kit (Abcam, ab229384), Human IL-6 ELISA kit (Abcam, ab178013) and Human tumor necrosis factor (TNF)-α ELISA kit (Abcam, ab181421) were used for the detection of the levels of IL-1β, IL-6 and TNF-α in the cell supernatant, respectively, in line with the manufacturer’s instruction.

### Immunofluorescence

Cells were seeded onto round-glass slides. After the adhesion, cells were fixed with 4% paraformaldehyde. Then, Triton X-100 (Beyotime, China) was used to enhance the permeability of cell membranes. After that, these cells were incubated with the primary antibody against gasdermin D (GSDMD; Abcam, ab219800) at 4°C overnight. Next, the fluorescent secondary antibody was applied for the subsequent incubation, which lasted for 2 hours at room temperature. Finally, the DAPI (Invitrogen, USA) was adopted to stain nucleus followed by the observation with the laser scanning confocal microscope (Olympus, Japan).

### Western blotting

The RIPA buffer (Beyotime, China) was utilized to extract protein samples. Then, the concentration of these protein samples was determined with the BCA kits (Beyotime, China). Next, these proteins underwent the separating procedure in 10% SDS-PAGE gel. After that, these proteins were transferred to the polyvinylidene fluoride (PVDF) membranes (Millipore, USA). The PVDF membranes were blocked with 5% skimmed milk at room temperature for 2 h and incubated with primary antibodies at 4°C overnight. The primary antibodies used were Ki-67 (Abcam, ab16667), HMGB1 (Abcam, ab18256), RAGE (Abcam, ab216329), Bcl-2 (Abcam, ab32124), Bax (Abcam, ab32503), Cleaved caspase3 (Abcam, ab32351), cyclin D1 (Abcam, ab16663), TLR4 (Abcam, ab13556), NF-κB p65 (Abcam, ab207297), NF-κB (Abcam, ab281518), caspase4 (Abcam, ab25898), NLRP3 (Abcam, ab263899) and GAPDH (Abcam, ab8245). On the following day, the membranes were incubated with peroxidase-conjugated secondary antibody. Finally, the immunoreactive signals were detected with the Pierce ECL Western Blotting Substrate (Thermo Fisher Scientific, USA).

### Statistical analysis

All the data in this study were analyzed with the Graphpad Prism 6.0. Each experiment was repeated for three times and the experimental data were displayed as mean ± SD. The data comparison was performed with the one-way ANOVA followed by Tukey’s post hoc test. The difference was considered as statistically significant until the P-value was less than 0.05.

## Results

### Stimulation of LPS induced the inflammation and apoptosis of cervical epithelial cells

**Figure 1.**
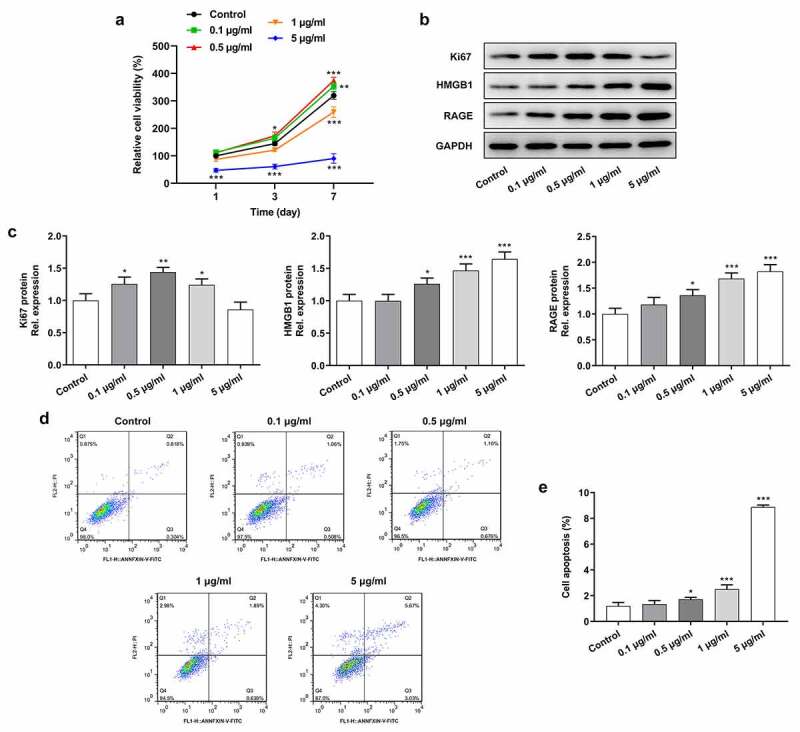
Stimulation of LPS affected the proliferation and apoptosis of cervical epithelial cells. (a) CCK-8 was used for the detection of the proliferation of cervical epithelial cells. (b, c) The expression of Ki-67, HMGB1 and RAGE in these cells was detected with the western blotting. (d, e) The apoptosis rates of these cells were determined with the flow cytometry. *p < 0.05, **p < 0.01, ***p < 0.001 vs control

To mimic chronic inflammation environment of cervical epithelial cells, diverse concentrations (0.1 μg/mL, 0.5 μg/mL, 1 μg/mL and 5 μg/mL) of LPS were used for the stimulation of cervical epithelial cells. Then, cell proliferation ability was detected by CCK-8 assay. It was indicated that low concentrations (0.1 μg/mL and 0.5 μg/mL) of LPS enhanced the proliferation of cervical epithelial cells while higher concentrations (1 μg/mL and 5 μg/mL) played an inhibiting role (Figure 1a). Next, the western blotting was employed for the detection of inflammation- and proliferation-related proteins. Figure 1b and c illustrated that the expression of Ki-67, HMGB1 and RAGE was promoted in these cells after the treatment of LPS. Furthermore, LPS induction at 0.5 μg/mL led to a slight increase of cell apoptosis, but LPS induction at 0.5 and 1 μg/mL caused significant increase of the apoptosis of cervical epithelial cells (Figure 1d and e).

### Inhibition of the expression of RAGE abolished the effect of LPS on the proliferation and migration of cervical epithelial cells

**Figure 2.**
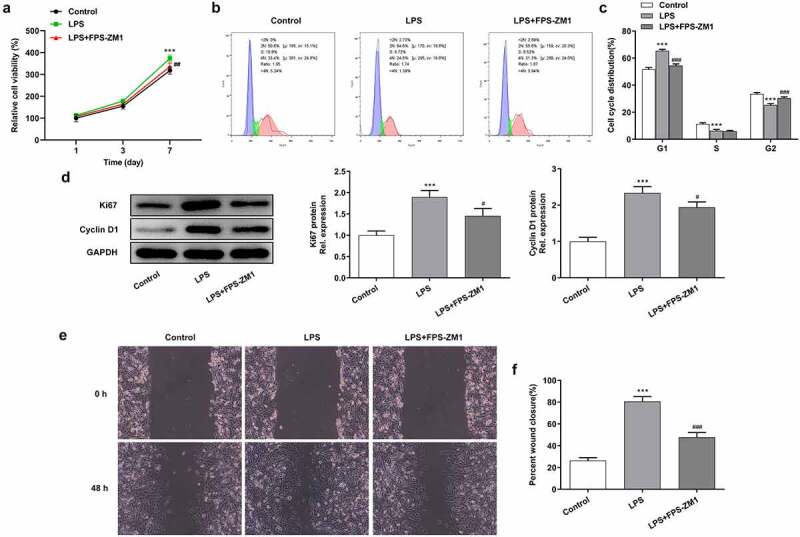
Inhibition of RAGE suppressed the proliferation and migration of cervical epithelial cells. (a) The proliferation of cervical epithelial cells was determined with the CCK-8. (b, c) The cell cycle was detected with the flow cytometry. (d) The expression of Ki-67 and cyclin D1 in cervical epithelial cells was detected with the western blotting. (e, f) The migration of cervical epithelial cells was determined with the wound healing assays. ***p < 0.001 vs control; ^#^p < 0.05, ^###^p < 0.001 vs LPS

To explore the role of HMGB1/RAGE in LPS-induced chronic inflammation of cervical epithelial cells, we selected the 0.5 μg/mL LPS for the subsequent assays, and the inhibitor of RAGE (FPS-ZM1, 25 nmol) was used for the suppression of the expression of RAGE in these cells. In order to determine the proliferative abilities of these cells, CCK-8 experiments were performed. Results (Figure 2a) showed that the stimulation of LPS promoted the proliferation of cervical epithelial cells while the proliferation was suppressed after the inhibition of RAGE. Then, the flow cytometry assay was adopted to determine the cell cycle. The data were presented in Figure 2b and c, which showed that application of RAGE inhibitor induced the arrest of G2/M stage and therefore restricted the proliferation of cervical epithelial cells. The following western blotting analysis was performed to detect the expression of Ki-67 and cyclin D1 in these cells. It was testified that stimulation of LPS promoted the expression of Ki-67 and cyclin D1 while the inhibition of RAGE suppressed the expression of these proteins in cervical epithelial cells (Figure 2d). After the western blot analysis, we observed the migration of these cells with the wound healing assay. Results (Figure 2e and f) showed that the stimulation of LPS enhanced the migration of these cells whereas application of RAGE inhibitor had an opposite effect.

### Application of RAGE inhibitor relieved the LPS-induced apoptosis of cervical epithelial cells

**Figure 3.**
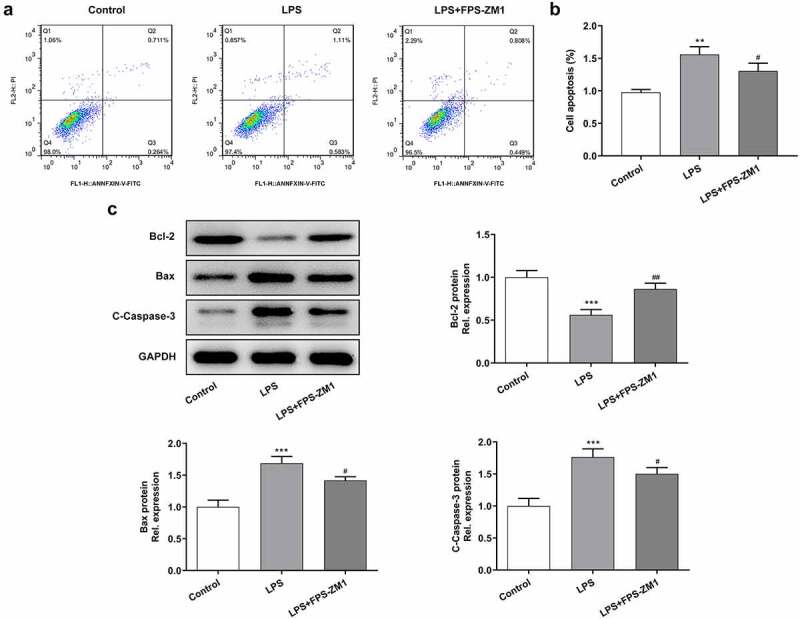
Suppression of RAGE relieved the LPS induced apoptosis of cervical epithelial cells. (a, b) The apoptosis of cervical epithelial cells was determined with the flow cytometry. (c) The expression of apoptosis related proteins was detected with the western blotting. **p < 0.01, ***p < 0.001 vs control; ^#^p < 0.05, ^##^p < 0.01 vs LPS

Then, Flow cytometry was performed to detect the apoptosis of cervical epithelial cells. It can be found from the results (Figure 3a and b), that LPS promoted the apoptosis of cervical epithelial cells while this apoptotic effect could be relieved by the RAGE inhibitor. The expression of apoptosis-related proteins also showed that the levels of Bcl-2 were increasing while the expressions of Bax and Cleaved caspase-3 were inhibited after the application of the inhibitor of RAGE (Figure 3c).

### Application of RAGE inhibitor alleviated the LPS-induced inflammation and pyroptosis of cervical epithelial cells

**Figure 4.**
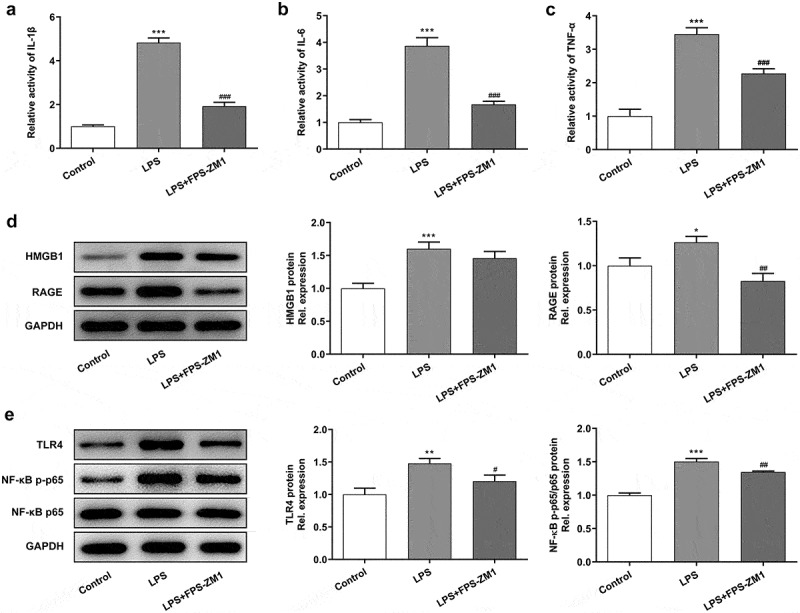
Inhibition of RAGE relieved the LPS induced inflammation of cervical epithelial cells. (a, b, c) The expression of inflammatory factors (IL-1β, IL-6 and TNF-α) was detected with the ELISA. (d) The expression of HMGB1 and RAGE in cervical epithelial cells was detected with the western blotting. (e) The expression of TLR4 and NF-κB p65 in cervical epithelial cells was determined with the western blotting. **p < 0.01, ***p < 0.001 vs control; ^#^p < 0.05, ^##^p < 0.01, ^###^p < 0.001 vs LPS

**Figure 5.**
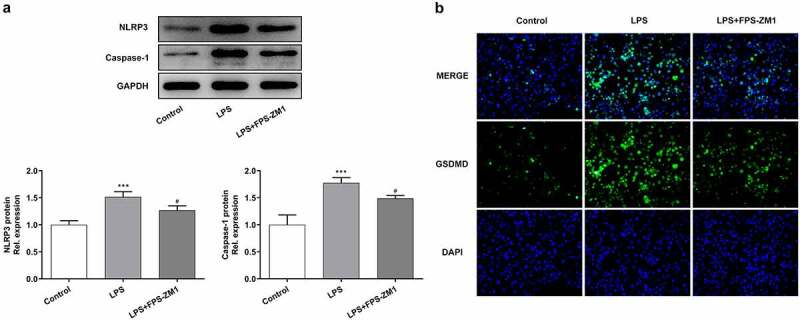
Suppression of RAGE relieved the pyroptosis of cervical epithelial cells. (a) The expression of NLRP3 and caspase4 in cervical epithelial cells was determined with the western blotting. (b) The GSDMD in cervical epithelial cells was observed with the immunofluorescence. ***p < 0.001 vs control; ^#^p < 0.05 vs LPS

Next, the effect of RAGE on LPS-induced chronic inflammation in cervical epithelial cells, as well as the potential molecular mechanism, was explored. In this part, ELISA assays were performed to detect the levels of inflammatory factors in these cells. The results (Figure 4a–c) showed that the expression of IL-1β, IL-6 and TNF-α was enhanced in cervical epithelial cells after the stimulation of LPS while the levels of these factors were decreased after the treatment of RAGE inhibitor. Next, western blotting identified that the using of LPS enhanced the expression of HMGB1 and RAGE. After treated with RAGE inhibitor, levels of these proteins presented a decreasing trend (Figure 4d). Moreover, the expression of TLR4 and NF-κB p-p65 also showed the same tendency (Figure 4e). Furthermore, the occurrence of the inflammation could induce the pyroptosis of multiple types of cells [15]. Therefore, we tended to identify how pyroptosis-related proteins express in cervical epithelial cells. The results of western blotting revealed that the stimulation of LPS promoted the expression of NLRP3 and caspase4 in these cells while the application of RAGE inhibitor had an inhibiting effect (Figure 5a). Finally, the pyroptosis symbol protein GSDMD was also observed with the immunofluorescence. Results indicated that LPS induced the increasing of the expression of GSDMD in these cells. In contrast, the inhibitor of RAGE repressed the expression of GSDMD in cervical epithelial cells (Figure 5b).

## Discussion

As a malignant tumor in gynecology, cervical cancer plays a pivotal role in female mortality (caused by cancer) worldwide [14]. Likewise, in most cases, women living in rural areas of China were deprived of life by this cancer [16]. Although the human papillomavirus vaccine has been widely used, the incidence of cervical cancer still shows a mounting tendency and the mortality is rising as well. Therefore, cervical cancer remains a major threat to human health [17]. At present, the main clinical treatment for cervical cancer is still surgical resection [18]. However, due to the strong metastatic and proliferative nature of cervical cancer cells, patients suffer from unfavorable prognosis after surgery. What’s more, the low survival rate within 5 years cannot be ignored [19]. Therefore, it is urgent for us to further probe into the molecular mechanism of the occurrence and development of cervical cancer, in which case, new therapeutic options might be found to improve the clinical treatment.

In addition, chronic inflammation is a crucial promoting factor during the occurrence and development of cancer [20]. Inflammation induced by diverse factors, including vaginal microbiologic disorder, could drive the cancer to occur [21,22]. Furthermore, HMGB1/RAGE axis was seen to be crucial during the development of the inflammation [13,23], and activation of HMGB1/RAGE axis could also promote the development of various sorts of cancer by inducing inflammation [10]. Studies also suggested that the signal path of HMGB1/RAGE could cause colorectal cancer to happen by activating the YAP1 [24], and the higher levels of HMGB1 and RAGE could also give rise to glioma [25]. In this study, we also found that the expression of HMGB1 and RAGE in cervical epithelial cells was enhanced during the process of LPS-induced chronic inflammation. Furthermore, the proliferation and migration of these cells were also promoted after the stimulation of LPS. However, the application of RAGE inhibitor not only put suppressive effects on the proliferative and migrative abilities of cervical epithelial cells, but also alleviated the LPS-induced inflammation. These results indicated that the suppression of RAGE abolished the LPS-induced inflammation as well as vicious transformation of cervical epithelial cells.

Moreover, the chronic inflammatory response could also induce the pyroptosis (a form of programmed cell death) of multiple types of cancer [26]. Studies have suggested that the pyroptosis induced by small molecules could suppress the proliferation of multiple types of tumor cells [27]. However, we found that the stimulation of LPS promoted the expression of pyroptosis-related proteins (NLRP3, caspase4 and GSDMD), but the application of RAGE inhibitor repressed the expression of these proteins in cervical epithelial cells. The results implied that the restriction of HMGB1/RAGE axis could also relieved the LPS-induced inflammation and pyroptosis.

## Conclusion

Above all, we determined the efficacy of HMGB1/RAGE axis on the vicious transformation of LPS-induced cervical epithelial cells. All the results suggested that inhibition of HMGB1/RAGE axis suppressed the LPS-induced vicious transformation of cervical epithelial cells. Our conclusion might provide a new target for the clinic treatment of cervical cancer.

## Highlights

Inhibition of HMGB1/RAGE axis suppressed the chronic inflammation of cervical epithelial cells.

Pyroptosis plays a critical role during vicious transformation of cervical epithelial cells.

Inhibition of HMGB1/RAGE suppressed LPS-induced cell proliferation.
